# Endothelial Microsomal Prostaglandin E Synthetase-1 Upregulates Vascularity and Endothelial Interleukin-1β in Deteriorative Progression of Experimental Autoimmune Encephalomyelitis

**DOI:** 10.3390/ijms19113647

**Published:** 2018-11-19

**Authors:** Takako Takemiya, Marumi Kawakami, Chisen Takeuchi

**Affiliations:** 1 Medical Research Institute, Tokyo Women’s Medical University, Tokyo 162-8666, Japan; kawakami.marumi@twmu.ac.jp; 2 Department of Neurology, Tokyo Metropolitan Kita Medical and Rehabilitation Center for the Disabled, Tokyo 114-0033, Japan; chisentakeuchi@gmail.com

**Keywords:** vascular endothelial cells, microsomal prostaglandin E synthetase-1, interleukin-1β, prostaglandin E_2_, experimental autoimmune encephalomyelitis, multiple sclerosis

## Abstract

Microsomal prostaglandin E synthetase-1 (mPGES-1) is an inducible terminal enzyme for the production of prostaglandin E_2_ (PGE_2_). In experimental autoimmune encephalomyelitis (EAE), an animal model of multiple sclerosis, mPGES-1 is induced in vascular endothelial cells (VECs) around inflammatory foci and facilitates inflammation, demyelination, and paralysis. Therefore, we investigated the role of CD31-positive VECs in mPGES-1-mediated EAE aggravation using immunohistochemical analysis and imaging of wild-type (wt) and *mPGES*-*1*-deficient (*mPGES-1*^−/−^) mice. We demonstrated that EAE induction facilitated vascularity in inflammatory lesions in the spinal cord, and this was significantly higher in wt mice than in *mPGES-1*^−/−^ mice. In addition, endothelial interleukin-1β (IL-1β) production was significantly higher in wt mice than in *mPGES-1*^−/−^ mice. Moreover, endothelial PGE_2_ receptors (E-prostanoid (EP) receptors EP1–4) were expressed after EAE induction, and IL-1β was induced in EP receptor-positive VECs. Furthermore, IL-1 receptor 1 expression on VECs was increased upon EAE induction. Thus, increased vascularity is one mechanism involved in EAE aggravation induced by mPGES-1. Furthermore, mPGES-1 facilitated the autocrine function of VECs upon EP receptor induction and IL-1β production, modulating mPGES-1 induction in EAE.

## 1. Introduction

Multiple sclerosis (MS) is a progressive central nervous system disease characterized by multifocal areas of leukocyte infiltration and demyelination. Inflammation is a key feature of MS that directly affects tissue injury. Inducible prostaglandin E_2_ (PGE_2_) is an inflammatory mediator that is sequentially synthesized from arachidonic acid by cyclooxygenase-2 (COX-2) and microsomal prostaglandin E synthase-1 (mPGES-1, also known as PTGES). PGE_2_ exerts its function by acting on four G-protein-coupled receptors (GPCRs) known as E-prostanoid (EP) receptors, including EP1, EP2, EP3, and EP4, with multiple isoforms of EP3.

mPGES-1 is induced in brain vascular endothelial cells (VECs) to mediate endotoxin-induced fever [1] and neuronal injury elicited by kainic acid (KA) [2]. COX-2 is also upregulated in macrophages/microglia in the brains of patients with MS [3] and in brain VECs in experimental autoimmune encephalomyelitis (EAE), an animal model of MS [4]. Rodent EAE induced by myelin oligodendrocyte glycoprotein 35–55 peptide (MOG_35–55_) is associated with inflammatory foci and typical perivascular infiltration of mononuclear cells in the spinal cord and brain [5,6].

Spinal expression of PGE_2_ is increased after EAE induction [7,8,9,10], and treatment with selective COX-2 inhibitors suppresses the development of EAE paralysis [11,12,13]. Similarly, mPGES-1 induction in infiltrating macrophages facilitates the clinical progression of EAE in mice [9]. In addition, PGE_2_ produced by endothelial mPGES-1 around inflammatory foci facilitates inflammation and demyelination in the spinal cord and aggravates paralysis in EAE [10]. 

Interleukin-1β (IL-1β) is a critical inflammatory and pathological mediator in the EAE spinal cord. Administration of recombinant interleukin-1 receptor antagonist (IL-1RN) delays disease onset and reduces EAE severity in rats [14]. Additionally, expression of a defective interleukin-1 receptor 1 (*IL-1R1*) gene in mice is associated with complete resistance to EAE [15]. IL-1β has been shown to facilitate PGE_2_ production. Conversely, we have demonstrated that PGE_2_ synthesized by mPGES-1 in VECs and macrophages/microglia facilitates IL-1β autocrine function in CD4-positive (CD4^+^) T cells and stimulates an intercellular interaction between CD4^+^ T cells in EAE development [16]. However, we found that IL-1β is increased in other regions, such as blood vessels in inflammatory lesions, in EAE. 

Accordingly, in this study, we investigated the roles of CD31-positive (CD31^+^) VECs in mPGES-1-mediated EAE aggravation through immunohistochemical analysis of *mPGES*-*1*-deficient (*mPGES-1*^−/−^, also known as *Ptges*^−/−^*)* mice. In particular, we investigated whether vascularity and endothelial IL-1β production were upregulated after EAE induction and were facilitated by PGE_2_ synthesized by mPGES-1. 

## 2. Results

### 2.1. mPGES-1 Was Induced in CD31^+^ VECs and Facilitated Demyelination and Paralysis

**Figure 1:**
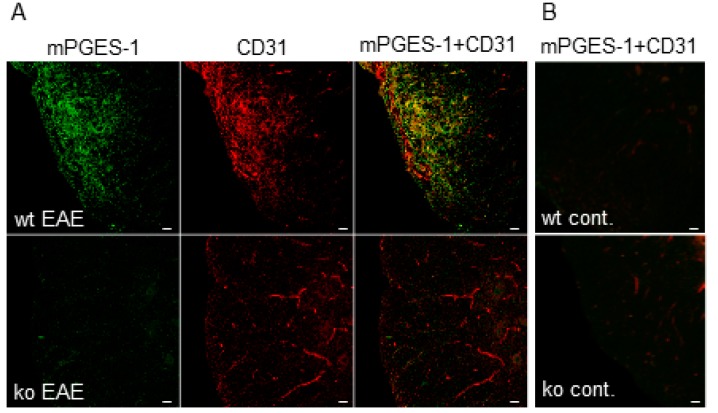
Expression of microsomal PGE synthase-1 (mPGES-1) in CD31-positive (CD31^+^) endothelial cells (ECs). Immunohistochemical staining of mPGE-1 (green) with CD31 (red) in the inflammatory regions of spinal cords of wild-type (wt) experimental autoimmune encephalomyelitis (EAE) mice and *mPGES-1*-deficient (*mPGES-1*^−/−^) EAE mice (**A**) and in the spinal cords of wt control mice and *mPGES-1*^−/−^ control mice (**B**). Scale bars, 20 μm (for all images); wt, wild-type; ko, *mPGES-1*^−/−^; cont., naïve mice; EAE, EAE mice.

According to immunohistochemical experiments, CD31^+^ ECs were increased and mPGES-1 was expressed in CD31^+^ ECs in the spinal cords of wild-type (wt) EAE mice (Figure 1A, upper panels). In contrast, only a slight increase in CD31^+^ ECs was observed and mPGES-1 was not expressed in mPGES-1^−/−^ EAE mice (Figure 1A, lower panels). We did not detect mPGES-1 in either of the controls (Figure 1B). This suggested that mPGES-1 expression was related to vascularity.

**Figure 2:**
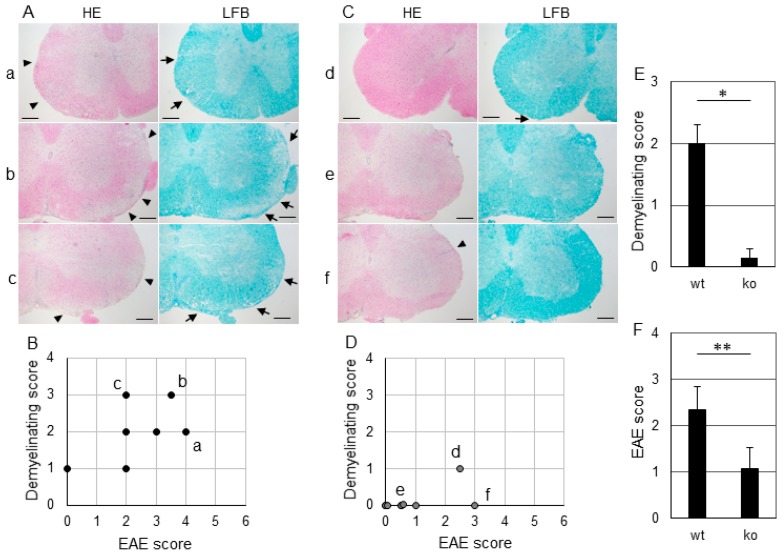
Histopathology of EAE mouse spinal cords at 19 days after immunization. Sections of lumbar spinal cord from wild-type (wt) experimental autoimmune encephalomyelitis (EAE) mice (**A**). Hematoxylin–eosin H–E staining showed massive infiltration of mononuclear cells into the parenchyma (left panel, arrowhead), and luxol fast blue (LFB) staining showed the diffuse demyelination (right panel, arrow) in wt EAE mice. Relationship between paralysis score and demyelinating score in wt EAE mice (**B**). Points a, b, and c in (**B**) corresponded to each panel in (**A**). Sections of lumbar spinal cord from microsomal PGE synthase-1 (mPGES-1)-deficient (*mPGES-1*^−/−^) EAE mice (**C**). H–E staining showed that infiltration was localized to the intravascular or leptomeningeal space (**left** panel, arrowhead), and LFB staining showed little demyelination (**right** panel, arrow). Relationship between paralysis score and demyelinating score in *mPGES-1*^−/−^ EAE mice (**D**). Points d, e, and f in (**D**) corresponded to each panel in (**C**). Demyelinating score in wt EAE mice and *mPGES-1*^−/−^ EAE mice (**E**). Paralysis score in wt EAE mice and *mPGES-1*^−/−^ EAE mice (**F**). Scale bars, 200 μm (for all images); wt, wild-type; ko, *mPGES-1*^−/−^; H–E, hematoxylin–eosin, LFB, luxol fast blue. * *p* < 0.001, ** *p* < 0.05.

We investigated whether demyelination in the EAE spinal cord and EAE paralysis were regulated by mPGES-1. Histopathological examination showed a marked difference between wt EAE and *mPGES-1*^−/−^ EAE mice at 19 days after immunization (Figure 2A,C). In wt EAE mice, hematoxylin and eosin (H–E)-stained sections showed diffuse and extensive infiltration of the parenchyma of the spinal cord (Figure 2A left); however, *mPGES-1*^−/−^ EAE mice revealed localized inflammatory lesions restricted to the perivascular or subpial space (Figure 2C left). Moreover, luxol fast blue (LFB)-stained sections showed diffuse and extensive demyelination in wt EAE mice (Figure 2A right). In contrast, demyelination was restricted to the perivascular lesion in *mPGES-1*^−/−^ EAE mice (Figure 2C right). The demyelinating score and the EAE score of *mPGES-1*^−/−^ EAE mice were significantly lower than those in wt EAE mice (Figure 2E,F). The relationship between the demyelinating score and the EAE score is shown in Figure 2B,D. The demyelinating score was not reflective of the EAE score in *mPGES-1*^−/−^ EAE mice, while the two were correlated in wt EAE mice (Figure 2B,D).

### 2.2. CD31^+^ VEC Invasion in the EAE Spinal Cord Was Facilitated by mPGES-1

**Figure 3:**
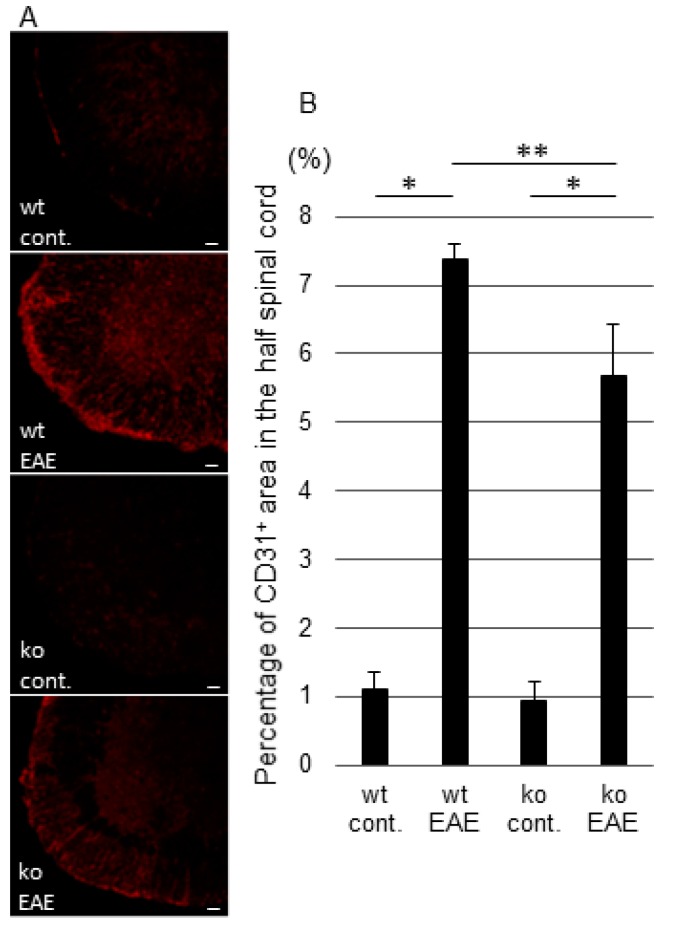
Distribution of CD31-positive (CD31^+^) endothelial cells (ECs) in spinal cords. Immunohistochemical image showing CD31 in the spinal cord in wild-type (wt) control, wt experimental autoimmune encephalomyelitis (EAE), *mPGES-1*-deficient (*mPGES-1*^−/−^) control, and *mPGES-1*^−/−^ EAE mice (**A**). Percentage of the CD31^+^ area in half of the spinal cord per half the size area of the spinal cord (**B**). Scale bar (50 μm); wt, wild-type; ko, *mPGES-1*^−/−^; cont., naïve mice; EAE, EAE mice. *p < 0.001, **p < 0.05.

We next investigated whether CD31^+^ EC invasion in the EAE spinal cord was regulated by mPGES-1. CD31^+^ ECs spread over the parenchyma in wt EAE mice; however, they were scattered in the spinal cord in mPGES-1^−/−^ EAE mice (Figure 3A). In addition, there were fewer CD31^+^ ECs in the control spinal cords of wt and mPGES-1^−/−^ mice (Figure 3A). These findings were also observed by spinal cord staining with tomato lectin (Figure S1). There were significant differences in percentage of the area stained for CD31 between wt control mice (1.1% ± 0.23%) and wt EAE mice (7.4% ± 0.23%; *p* < 0.001, Figure 3B) and between mPGES-1^−/−^ control mice (0.94% ± 0.27%) and mPGES-1^−/−^ EAE mice (5.7% ± 0.78%; *p* < 0.001, Figure 3B). Moreover, the percentage of the area stained for CD31 was significantly higher in wt EAE mice (7.4% ± 0.23%) than in mPGES-1^−/−^ EAE mice (5.7% ± 0.78%; *p* = 0.0345, Figure 3B). We found that vascularity in the spinal cord was increased upon EAE induction and by PGE_2_ synthesized by mPGES-1. 

### 2.3. IL-1β Upregulation in CD31^+^ VECs in EAE Spinal Cords Was Regulated by mPGES-1

**Figure 4:**
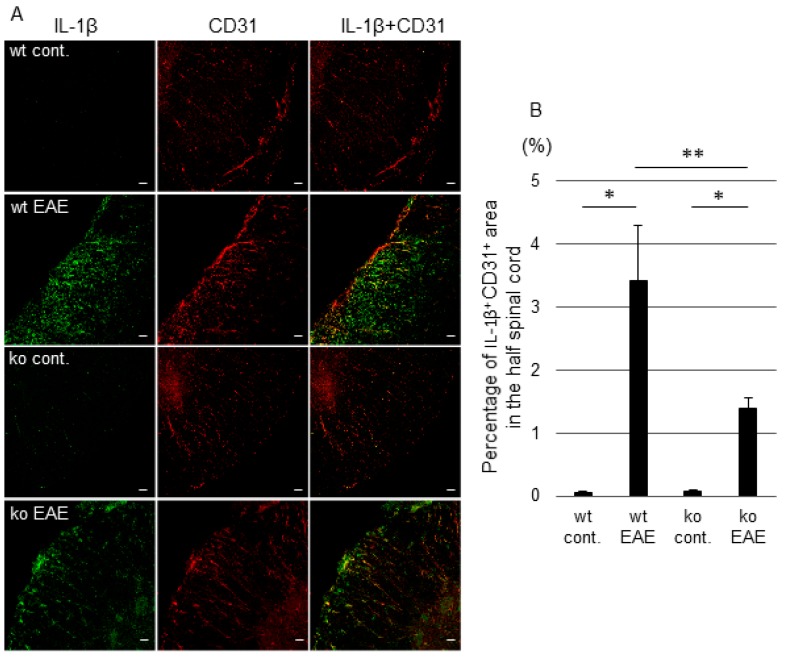
Expression of interleukin-1β (IL-1β) in CD31-positive (CD31^+^) endothelial cells (ECs) in the spinal cords of control and experimental autoimmune encephalomyelitis (EAE) mice. Immunohistochemical images showing IL-1β (green) and CD31 (red) in the spinal cords of wild-type (wt) control, wt EAE, microsomal PGE synthase-1-deficient (*mPGES-1*^−/−^) control, and *mPGES-1*^−/−^ EAE mice (**A**). Percentage of IL-1β-positive (IL-1β^+^) CD31^+^ area in half of the spinal cord per half of the spinal cord area (**B**). Scale bars are 20 μm; wt, wild-type; ko, *mPGES-1*^−/−^; cont., naïve mice; EAE, EAE mice. * *p* < 0.001, **p < 0.05.

We next studied whether IL-1β was upregulated in invaded CD31^+^ ECs. We found no IL-1β in either wt or *mPGES-1*^−/−^ control mice (Figure 4). In EAE mice, the percentage of the IL-1β-positive CD31^+^ area in half of the spinal cord per half of the spinal cord area was significantly higher in wt EAE mice (3.4% ± 0.88%) than in *mPGES-1*^−/−^ EAE mice (1.4% ± 0.16%; *p* = 0.0183, Figure 4), suggesting that IL-1β production may be regulated by mPGES-1-synthesized PGE_2_. 

### 2.4. Induction of EP Receptors in CD31^+^ VECs in the EAE Spinal Cord

**Figure 5:**
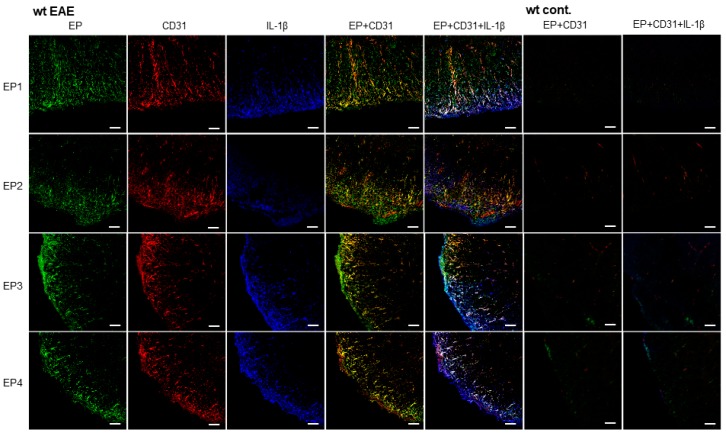
Expression of E-prostanoid (EP) receptors and interleukin-1β (IL-1β) in CD31-positive (CD31^+^) endothelial cells (ECs) in spinal cords in wild-type (wt) mice. Immunohistochemical images showing EP1–4 (green), CD31 (red), and IL-1β (blue) in the inflammatory region of the spinal cords of experimental autoimmune encephalomyelitis (EAE) wt mice (wt EAE) and in the naïve spinal cords in wt control mice (wt cont.). Scale bars are 50 μm (for all images); wt, wild-type; EAE, EAE mice; cont., naïve mice.

**Figure 6:**
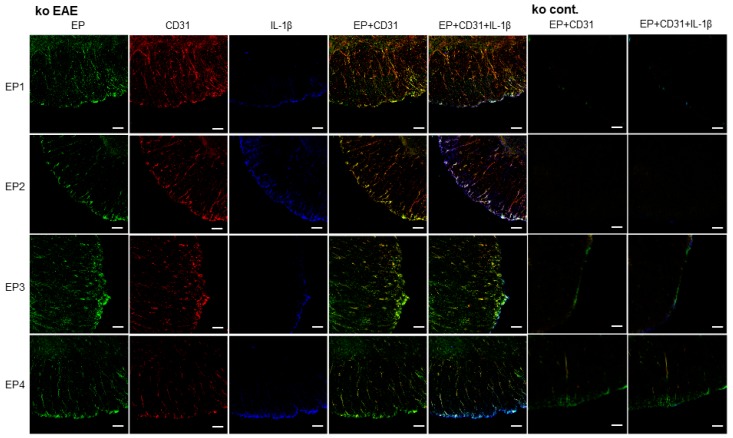
Expression of E-prostanoid (EP) receptors and interleukin-1β (IL-1β) in CD31-positive (CD31^+^) endothelial cells (ECs) in spinal cords in microsomal PGE synthase-1-deficient (*mPGES-1*^−/−^) mice. Immunohistochemical images showing EP1–4 (green), CD31 (red), and IL-1β (blue) in the inflammatory region of the spinal cords of experimental autoimmune encephalomyelitis (EAE) *mPGES-1*^−/−^ mice (*mPGES-1*^−/−^ EAE) and in the naïve spinal cords in *mPGES-1*^−/−^ control mice (*mPGES-1*^−/−^ cont.). Scale bars are 50 μm (for all images). ko, *mPGES-1*^−/−^; EAE, EAE mice; cont., naïve mice.

The above findings raised the question of whether PGE_2_ activated the PGE_2_ receptors EP1–4 on ECs to facilitate vascularity and IL-1β production. Therefore, we next investigated the expression of EP1–4 on ECs. EP receptors 1–4 were strongly stained and widely colocalized with CD31 in the spinal cords of wt EAE mice (Figure 5), but only weakly stained and partially colocalized in mPGES-1^−/−^ EAE mice (Figure 6). In addition, there was no expression of EP receptors in control spinal cords, regardless of the presence of mPGES-1 (Figure 5 and Figure 6). The expression and distribution of endothelial EP receptors were quantitatively affected by the increase in CD31^+^ ECs in both groups. This suggested that EP receptor expression was increased according to the increase in ECs upon EAE induction. In addition, partial IL-1β staining was observed in EP-positive CD31^+^ ECs in wt EAE mice (Figure 5). Furthermore, slight EP-positive EC staining for IL-1β was observed in mPGES-1^−/−^ EAE mice (Figure 6). These findings suggested that endothelial IL-1β production is stimulated by PGE_2_ through EP receptor activation.

### 2.5. IL-1R1 Upregulation in CD31^+^ VECs in EAE Spinal Cords Was Not Regulated by mPGES-1

**Figure 7:**
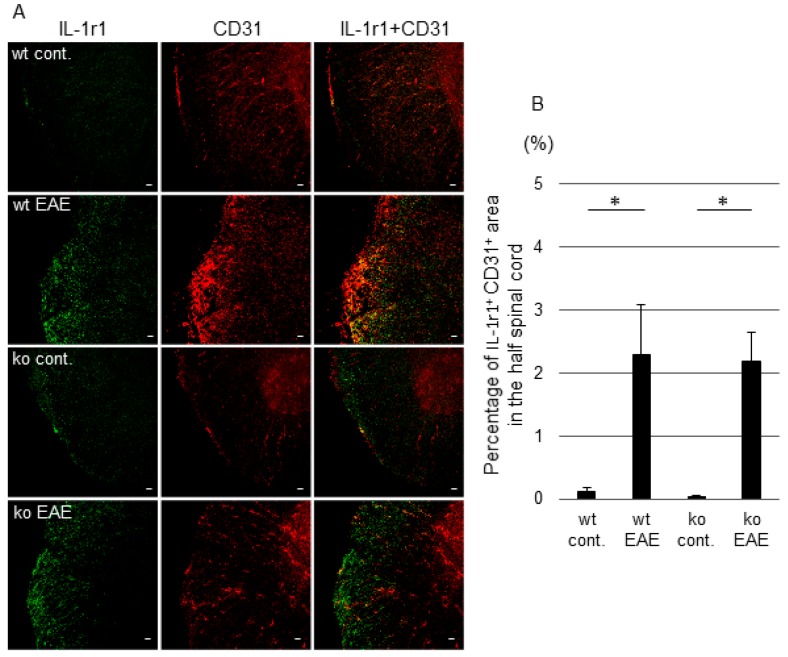
Expression of IL-1 receptor 1 (IL-1R1) in CD31-positive (CD31^+^) endothelial cells (ECs) in the spinal cords of control and experimental autoimmune encephalomyelitis (EAE) mice. Immunohistochemical images showing IL-1R1 (green) and CD31 (red) in the spinal cords of wild-type (wt) control, wt EAE, microsomal PGE synthase-1 (*mPGES-1*)^−/−^ control, and *mPGES-1*^−/−^ EAE mice (**A**). Percentage of the IL-1R1-positive (IL-1R1^+^) CD31^+^ area in half of the spinal cord per half of the spinal cord area (**B**). Scale bars are 20 μm; wt, wild-type; ko, *mPGES-1*^−/−^; cont., naïve mice; EAE, EAE mice. * *p* < 0.05.

IL-1R1 was not expressed in control spinal cords, regardless of the presence of mPGES-1, and IL-1R1 staining in ECs was weak and consistent in the spinal cords of wt EAE mice and mPGES-1^−/−^ EAE mice (Figure 7A). There were significant differences in the percentages of IL-1R1-positive (IL-1R1^+^) CD31^+^ areas in half of the spinal cord per of the half spinal cord area between wt control and wt EAE mice and between mPGES-1^−/−^ control and mPGES-1^−/−^ EAE mice (Figure 7B, *p* < 0.05). However, there were no significant differences in the percentages of IL-1R1^+^ CD31^+^ areas in half of the spinal cord per of the half spinal cord area between wt EAE mice (2.3% ± 0.79%) and mPGES-1^−/−^ EAE mice (2.2% ± 0.47%; *p* = 0.4523, Figure 7B).

## 3. Discussion

### 3.1. Vascularity and mPGES-1 Expression in EAE

In EAE spinal cords, blood-brain barrier (BBB) disruption and subsequent infiltration of immune cells, such as T cells or macrophages, were observed in inflammatory lesions and were found to be regulated by matrix metalloproteinases (MMPs) [17,18]. MMPs are known to contribute to the neuro-inflammatory response in many neurological diseases, and induction of MMP-2 is mediated by the binding of vascular cell adhesion molecule-1 on VECs to the very late activation-4 antigen expressed on T cells and macrophages [19]. Therefore, blood vessels play an important role in EAE pathology. Moreover, inflammation induces the vasodilation of small blood vessels, allowing for more perfusion and resulting in a distinct increase in vessel density and vessel immunostaining. After EAE induction, the blood vessel density, as determined by staining with CD31 to label ECs, is increased because of vasodilation and angiogenesis [20]. Angiogenesis is stimulated by vascular endothelial growth factor (VEGF), which leads to degeneration of the vascular basement membrane and breakdown of the BBB [21]. BBB dysregulation and transendothelial migration of activated leukocytes are the earliest cerebrovascular dysfunctions in MS [22]. VEGF is involved in EAE progression [20] during the acute phase (day 21) but not the chronic phase (day 55) [23]. In addition, inhibition of VEGF receptor 2 decreases clinical signs of EAE in the acute phase of the EAE process [23]. Moreover, the number of blood vessels increases during the EAE relapse phase [20]; thus, angiogenesis aggravates inflammation in the spinal cord in EAE. In contrast, angiogenesis induced after inflammation promotes neuronal remodeling through vessel-derived prostacyclin (prostaglandin I_2_) during the chronic phase of EAE [24,25]. In our study, sections of mouse spinal cord tissues on day 19 after EAE induction were stained for CD31 to compare the blood vessel densities of wt EAE mice and *mPGES-1*^−/−^ EAE mice. CD31^+^ ECs were increased in EAE spinal cords. We also used tomato lectin, which is an effective marker of blood vessels and microglial cells in rodents. Tomato lectin staining was also increased in EAE mice. EAE induction significantly increased CD31^+^ ECs in both wt and *mPGES-1*^−/−^ mice compared with those in the corresponding normal control groups. In addition, there was a significant difference in the increase in CD31^+^ ECs between wt and *mPGES-1*^−/−^ EAE mice, but no significant difference was observed between normal control wt and *mPGES-1*^−/−^ mice. These results suggest that vascularity is induced upon EAE induction, regardless of the presence of mPGES-1. Furthermore, the PGE_2_ produced by mPGES-1 facilitates increased vascularity in the EAE spinal cord. 

Leukocyte cultures from patients with definite MS show higher PGE expression than those from healthy controls [26]. Moreover, PGE_2_ levels are higher in peripheral blood monocytes from patients with chronic progressive MS [27]. These findings suggest that PGE_2_ is related to MS development. Inducible PGE_2_ is sequentially synthesized from arachidonic acid by COX-2, which is induced in chronic active lesions, specifically near damaged oligodendrocytes in macrophages and microglia in patients with MS [3]. Because COX-2 is localized to von-Willebrand factor-marked ECs in the spinal cord in EAE, particularly at 14–25 days after immunization [4], the PGE_2_ produced in this period plays some role in EAE lesions. PGES has three isoforms, i.e., mPGES-1 [28], microsomal prostaglandin E synthase-2 (mPGES-2) [29], and cytosolic PGE_2_ synthase (cPGES) [30]. mPGES-2 and cPGES are constitutively expressed, whereas mPGES-1 is induced following stimulation with pro-inflammatory factors and is functionally coupled with COX-2 [31]. In the brain, mPGES-1 is induced in VECs in fever [1] or KA-elicited neuronal damage [2]. In EAE, mPGES-1 appears in macrophages [9] and ECs in the spinal cord 19 days after immunization [10]. We confirmed that mPGES-1 was expressed in CD31^+^ ECs and CD11^+^ macrophages/microglia on day 19 after immunization but was not expressed in CD4^+^ T cells [16]. In this study, we also confirmed that mPGES-1 was strongly induced in CD31^+^ ECs. Upregulation of vascularity is associated with an increase in the area in which mPGES-1 is produced. 

### 3.2. Effect of IL-1β on EAE and Endothelial IL-1β Production

Several studies have shown that IL-1β plays a crucial role in EAE. Administration of recombinant IL-1RN to EAE rats delays the onset of the disease and reduces its severity [14]. Moreover, a defective *IL-1R1* gene in mice is associated with complete resistance to EAE [15]. Recently, it was reported that IL-1β is the main protagonist of EAE, whereas IL-1α is dispensable [32]. IL-1β regulates BBB permeability [33,34,35] and induces BBB disruption [36]. IL-1β also exacerbates neuro-inflammation [37] and is highly expressed in infiltrating macrophages [38]. *IL-1β* mRNA is induced in peritoneal leukocytes immediately after the initial induction of EAE [39]. Moreover, activated macrophages are thought to be the predominant source of IL-1β in the brain [40]. However, the contribution of macrophages to the total production of IL-1β is minor in EAE pathogenesis [32,38], and CD4^+^ T cells are thought to be a major source of IL-1β during EAE pathogenesis [41]. IL-1β stimulates ECs via IL-1R1 to produce cytokines, such as IL-6 and CXCL2, in inflammatory lesions in EAE [32]; however, no studies have evaluated the production of IL-1β in ECs. Therefore, in this study, we investigated whether IL-1β expression was upregulated in ECs by PGE_2_ synthesized from mPGES-1. We compared IL-1β expression in CD31^+^ ECs in wt and *mPGES-1*^−/−^ mice. IL-1β was rarely co-expressed with CD31 in normal control wt mice or *mPGES-1*^−/−^ mice. In contrast, IL-1β expression was significantly increased in CD31^+^ ECs in EAE mice. In addition, the percent area co-expressing IL-1β and CD31 per half of the spinal cord area was significantly higher in wt EAE mice than in *mPGES-1*^−/−^ mice. 

### 3.3. Expression and Effect of Endothelial EP Receptors

All four EP receptors are expressed in neurons and VECs under pathological conditions, such as hypoxic–ischemic encephalopathy [42]. In this study, there was low EP receptor expression in normal control wt mice and *mPGES-1*^−/−^ mice. In contrast, EP receptors 1–4 were strongly stained and widely colocalized with CD31 in the spinal cords of wt EAE mice but were weakly stained and only partially colocalized with CD31 in *mPGES-1*^−/−^ EAE mice. 

EP1 antagonists and genetic deletion of EP1 protect against BBB disruption following experimental ischemic stroke in rats and mice [43], and the activation of EP2 or EP4 induces vasodilation and hyperpermeability in modified Miles assays in mice [44]. Moreover, inhibition of EP3 or EP4 attenuates arachidonic acid-mediated permeability of human brain microvascular ECs [45]. These reports suggest that the activation of EP1–4 aggravates BBB disruption and increases BBB permeability. However, protective effects against permeability have also been reported upon activation of EP3 and EP4. EP3 activation has been reported to lead to hypopermeability [44], and endothelial deletion of EP4 worsens stroke injury and decreases cerebral reperfusion in a mouse model of cerebral ischemia [46]. Thus, activation of endothelial EP receptors may have opposing effects on BBB permeability depending on the timing and pathological conditions.

### 3.4. IL-1β Production Through EP Receptor Activation

EP receptor-positive (EP receptor^+^) CD4^+^ T cells almost entirely colocalized with IL-1β, which is regulated by PGE_2_ synthesized by mPGES-1 [16]. T cells are also regulated by the activation of EP2 or EP4 [47,48,49], and IL-1β upregulates EP2 and EP4 in primary cultured hippocampal neurons [50]. Thus, the activation of EP2 or EP4 may increase IL-1β production in CD4^+^ T cells, which controls the induction of EP receptors. In other words, the activation of CD4^+^ T cells is regulated by autocrine functioning via IL-1β and PGE_2_. In triple staining for EP receptors, CD31, and IL-1β in this study, IL-1β was detected in EP receptor^+^ CD31^+^ ECs in wt EAE mice; therefore, IL-1β expression in ECs may be stimulated by PGE_2_ via EP receptors. Furthermore, we cannot exclude the possibility that the PGE_2_ produced by other PGESs may activate EP receptors to produce IL-1β in ECs in *mPGES-1*^−/−^ EAE mice, as there were few EP receptor^+^ ECs stained with IL-1β in *mPGES-1*^−/−^ EAE mice.

### 3.5. Relationship between IL-1β and COX-2/mPGES-1

IL-1β is known to stimulate COX-2 induction. Intraperitoneal IL-1β injection induces the expression of *COX-2* mRNA in VECs in the brain [51] and leads to the induction of *COX-2* mRNA in cultured primary hippocampal neurons [52]. The main inducer of central COX-2 upregulation in the spinal cord is IL-1β, which thus promotes the production of PGE_2_ and contributes to inflammatory pain hypersensitivity [53]. IL-1β also has an essential role in mediating the activity of nuclear factor kappa B and the transcription of *COX-2* in cells of the BBB in inflammation [54]. In the brain, COX-2 inhibitors prevent IL-1β-induced increases in PGE_2_ production [55], suggesting that IL-1β regulates PGE_2_ production through the synthesis of COX-2. In addition, crosstalk between microvascular ECs and tumor cells upregulates COX-2 and mPGES-1, which are both strongly inhibited by an IL-1R antagonist [56]. Thus, IL-1β plays a crucial role in the induction of COX-2 and mPGES-1 to produce pathophysiological PGE_2_, which promotes inflammation. In contrast, we first demonstrated that IL-1β in CD4^+^ T cells was controlled by endothelial PGE_2_ derived from mPGES-1 in EAE spinal cords [16]. Therefore, endothelial IL-1β may also be regulated by endothelial PGE_2_ derived from mPGES-1 in EAE spinal cords as an autocrine function between neighboring ECs.

### 3.6. Expression of Endothelial IL-1R1

The *IL-1R1* and *IL-1R2* mRNAs are expressed in the ECs of rat brains [57], and knockdown of IL-1R1 in ECs attenuates stress-induced neuro-inflammation [58]. Endothelial IL-1R1 is an important receptor in EAE pathology [32,59], and IL-1R1 signaling controls expression of the chemokine CXCL12 at the BBB, which affects the severity of EAE [60]. In this study, IL-1R1 expression was significantly increased in CD31^+^ ECs in EAE mice; in contrast, IL-1R1 was rarely co-expressed with CD31 in normal control mice. However, there was no significant difference in IL-1R1 expression in CD31^+^ ECs between wt EAE mice and *mPGES-1*^−/−^ EAE mice or between wt control mice and *mPGES-1*^−/−^ control mice. These results suggest that mPGES-1 signaling may not control the induction of endothelial IL-1R1 in EAE. However, because IL-1β in CD4^+^ T cells is upregulated in wt EAE, EC activation could be increased by IL-1β produced in nearby CD4^+^ T cells in wt mice. Thus, further studies are necessary to assess the effects of IL-1R1 activation on vascularity. 

### 3.7. A Mechanism of EAE Aggravation Induced by mPGES-1

**Scheme 1:**
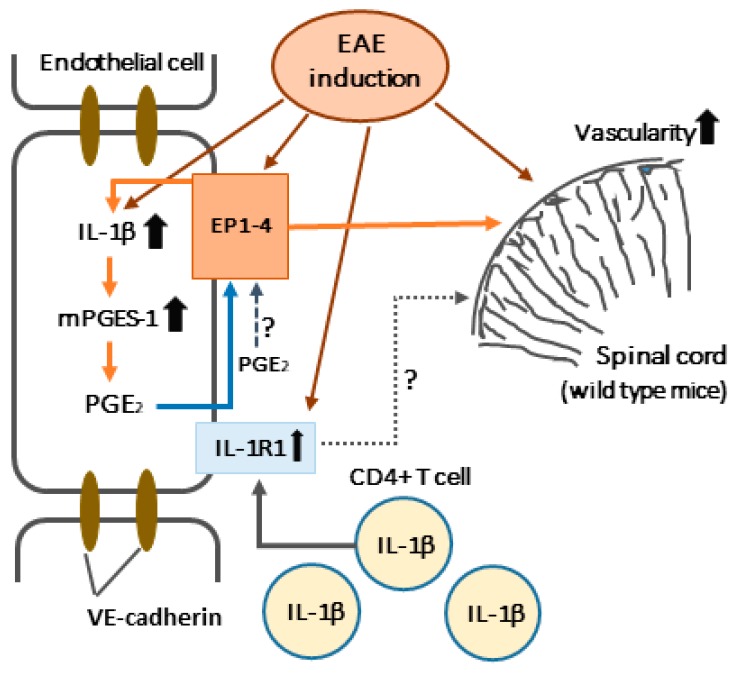
Role of vascular endothelial cells (VECs) in microsomal PGE synthase-1 (mPGES-1)-related aggravation of experimental autoimmune encephalomyelitis (EAE). Upregulation of vascularity is increased in EAE spinal cords in wild-type (wt) mice. In VECs, prostaglandin E_2_ (PGE_2_) is synthesized by mPGES-1 in response to stimulation by interleukin-1β (IL-1β). Expression of the endothelial PGE_2_ EP receptors is increased and IL-1β is induced in EP receptor-positive VECs in wt mice; therefore, activation of EP receptors may be related to IL-1β production in VECs. IL-1 receptor 1 (IL-1R1) is increased on VECs by EAE induction. IL-1β in CD4-positive (CD4^+^) T cells shows greater upregulation in wt EAE; thus, VECs may be activated by IL-1β produced in nearby CD4^+^ T cells in wt mice. VE-cadherin, vascular endothelial-cadherin.

**Scheme 2:**
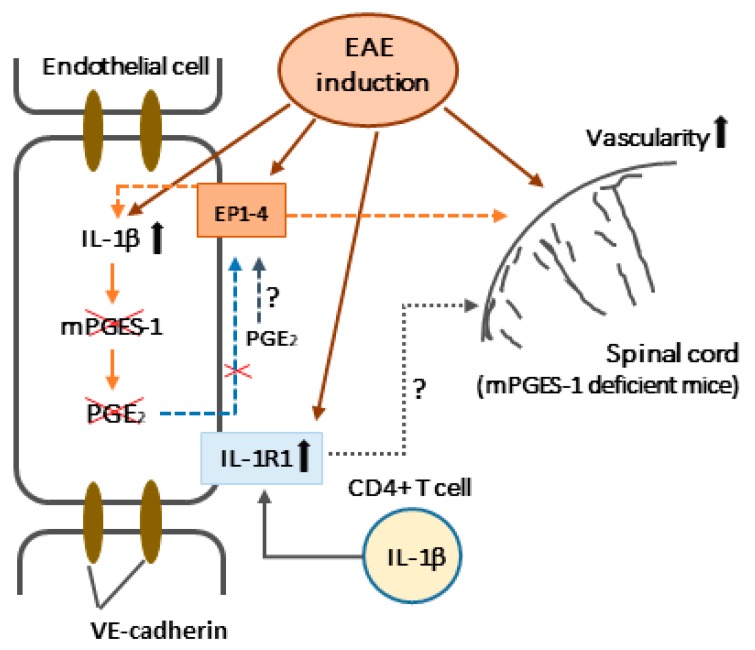
Effects of microsomal PGE synthase-1 (*mPGES-1*) deficiency on vascularity and interleukin-1β (IL-1β) production in vascular endothelial cells (VECs). Upregulation of vascularity is minimal in experimental autoimmune encephalomyelitis (EAE) spinal cords in *mPGES-1*-deficient (*mPGES-1*^−/−^) mice compared with that in wt mice. In VECs, PGE_2_ is not synthesized. Induction of the endothelial PGE_2_ EP receptors and production of IL-1β are decreased in *mPGES-1*^−/−^ mice compared with that in wt mice. IL-1 receptor 1 (IL-1R1) is upregulated on VECs by EAE induction, but there is less IL-1β in CD4-positive (CD4^+^) T cells in *mPGES-1*^−/−^ mice. Therefore, VECs may be slightly activated by IL-1β produced in nearby CD4^+^ T cells in *mPGES-1*^−/−^ mice. VE-cadherin, vascular endothelial-cadherin.

In this study, we demonstrated the upregulation of vascularity in EAE, which was regulated by mPGES-1 upon the induction of EP receptors, potentiation of IL-1β production, and expression of IL-1R1 on CD31^+^ ECs (Scheme 1). Moreover, IL-1β production may be increased by PGE_2_ through EP receptors in CD31^+^ ECs (Scheme 1). This signaling pathway is regulated by mPGES-1; therefore, mPGES-1-deficient mice showed weak vascularity with just a slight increase in EP receptor expression and IL-1β production (Scheme 2). The PGE_2_ produced by mPGES-1 aggravates inflammation and the progression of EAE [9,10,16]; thus, the upregulation of vascularity is an mPGES-1-mediated mechanism of EAE aggravation, as VECs are an important source of mPGES-1 in inflammatory foci. Furthermore, mPGES-1 facilitates the autocrine function of VECs upon EP receptor induction and increases IL-1β production, thereby facilitating mPGES-1 induction in EAE. 

## 4. Materials and Methods

### 4.1. Mice

We used seven female *mPGES-1*^−/−^ mice (8 weeks of age) and seven female age-matched C57BL/6J mice in this study. *mPGES-1*^−/−^ mice were generated as previously described [61]. We used untreated female wt mice and *mPGES-1*^−/−^ mice as normal controls. Seven mice were included in each group, and the mice were housed three or four per cage in a room maintained at 24 ± 2 °C with a standard 12-h light-dark cycle and with access to standard chow and water ad libitum. All animal experiments were approved by the Ethical Review Committee of Animal Experiments and Gene Recombination Experiment Safety Committee of Tokyo Women’s Medical University (14–91/14–44, 29 March 2014).

### 4.2. Induction and Assessment of EAE

Mice were immunized by subcutaneous injection with 250 μg MOG_35–55_ (MEVGWYRSPFSRVVHLYRNGK, purity >95%; Operon Technology, Tokyo, Japan) in complete Freund’s adjuvant (Difco, Detroit, MI, USA). The mice also received two intraperitoneal injections of 500 ng of pertussis toxin each (Seikagaku Corporation, Tokyo, Japan), once on the day of immunization and again 2 days later. The mice were observed daily, and we assessed the progression of EAE using the following scoring system: (0) No detectable signs of paralysis; (1) completely limp tail; (2) loss of the righting reflex; (3) partial hind limb paralysis; (4) complete hind limb paralysis; (5) total paralysis of all four limbs; and (6) death. Mice that were scored as 5 for two consecutive days were immediately euthanized.

### 4.3. Immunohistochemistry

The mice were sacrificed under deep anesthesia induced by an intraperitoneal injection of pentobarbital (120 mg/kg) on day 19 after immunization. Spinal cords were quickly removed and frozen with a dry ice/acetone mixture. Sections were cut at a thickness of 10 μm and left to air dry at room temperature for 30 min, after which they were rinsed with phosphate-buffered saline. Next, the sections were treated with 10% normal goat serum for 3–5 h, incubated with anti-mPGES-1 antibodies (Cayman Chemical, Ann Arbor, MI, USA; #160140; 1:250 dilution) overnight, and then incubated with anti-EP1 (Bioss, Woburn, MA, USA; bs-6316R; 1:100), anti-EP2 (Bioss; bs-4196R; 1:100), anti-EP3 (Santa Cruz Biotechnology, Inc., Dallas, TX, USA; sc-20676; 1:100), anti-EP4 (Bioss; bs-8538R; 1:100), anti-IL-1β (Santa Cruz Biotechnology, Inc.; sc-15325; 1:100; Cloud-Clone, USA; MAA563Mu21; 1:25), or anti-IL-1R1 (Santa Cruz Biotechnology, Inc.; sc-689; 1:100) antibodies for two nights at 4 °C with slow shaking. To visualize ECs, the sections were double stained with anti-CD31 (BD Bioscience, La Jolla, CA, USA; #550274; 1:20) antibodies. After removal of the primary antibody, the sections were incubated with fluorescein isothiocyanate (FITC)-labeled anti-rabbit IgG (1:150) to label mPGES-1, EP1–4, IL-1β, and IL-1R1 or with Cy3-labeled anti-rat IgG (1:150) to label CD31 for 5 h at room temperature with slow shaking. For visualization of blood vessels, sections were also incubated with FITC-labeled tomato lectin (1:200 dilution). Fluorescent images were obtained using a confocal microscope LSM510 and LSM710 (Carl Zeiss Microscopy GmbH, Jena, Germany).

### 4.4. Image Analysis

To analyze the fluorescent images, we measured the CD31-stained area in the inflammatory half of the spinal cord using the software ZEN2.3 (Carl Zeiss Microscopy GmbH, Jena, Germany) and then calculated the ratio of the CD31-stained area to the total half-spine area. In addition, we measured the IL-1β- or IL-1R1-stained area colocalized with CD31 in each experiment and calculated the ratio of the contained area to the CD31-stained area using the colocalization function of the software AIM (Carl Zeiss Microscopy GmbH, Jena, Germany).

### 4.5. Histopathology

The mice were sacrificed under deep anesthesia induced by the intraperitoneal injection of pentobarbital (120 mg/kg) on day 19 after immunization. The lumbar spinal cords were quickly removed and frozen. All of the spinal cords were embedded in optimal cutting temperature compound and frozen with a dry ice/acetone mixture. Subsequently, sections were cut at a thickness of 10 μm using a cryostat. The sections were stained with H–E, which stains inflammatory cells, and LFB, which stains myelin and myelinated axons. Myelinated fibers were stained blue, and the demyelinated area was pale in LFB-stained tissue. Images of the sections were captured using a microscope equipped with a digital camera (OLYMPUS BX-40, Tokyo, Japan). Demyelinating lesions were graded using LFB-stained sections as follows: Grade 1, trace levels of perivascular or subpial demyelination; Grade 2, focal demyelination; Grade 3, demyelination involving a quarter of the tissues examined; Grade 4, massive confluent parenchymal demyelination involving half of the tissue; and Grade 5, extensive demyelination involving the entire tissue [62,63].

### 4.6. Statistical Analysis

The data are presented as means ± standard errors. Statistical analysis was performed using independent Student’s *t*-tests with the assumption of equal variance, and significance was determined at a *p* value of less than 0.05.

## 5. Conclusions

We found that PGE_2_, synthesized by mPGES-1, upregulated vascularity, and endothelial IL-1β production through a PGE_2_ autocrine function in spinal cords in EAE. In this study, we did not evaluate the mechanisms of brain lesions in MS; however, the vascularity induced by this PGE_2_-synthesizing process in VECs may have an important role in neuro-inflammatory changes in neurodegenerative diseases.

## Supplementary Materials

The following are available online at http://www.mdpi.com/1422-0067/19/11/3647/s1.

## Abbreviations

MS	multiple sclerosis
PGE_2_	prostaglandin E_2_
COX-2	cyclooxygenase-2
mPGES-1	microsomal prostaglandin E synthetase-1
GPCRs	G-protein-coupled receptors
EP	E-prostanoid
VECs	vascular endothelial cells
KA	kainic acid
EAE	experimental autoimmune encephalomyelitis
MOG_35–55_	myelin oligodendrocyte glycoprotein_35–55_ peptide
IL-1β	interleukin-1β
IL-1RN	interleukin-1 receptor antagonist
IL-1R1	interleukin-1 receptor 1
CD4^+^ T cells	CD4-positive T cells
CD31^+^	CD31-positive
*mPGES-1* ^−/−^	microsomal prostaglandin synthetase-1-deficient
wt	wild-type
H–E	hematoxylin and eosin
LFB	luxol fast blue
IL-1R1^+^	IL-1R1-positive
BBB	blood-brain barrier
MMPs	matrix metalloproteinases
VEGF	vascular endothelial growth factor
mPGES-2	microsomal prostaglandin E synthase-2
cPGES	cytosolic PGE_2_ synthase
EP receptor^+^	EP receptor-positive
VE-cadherin	vascular endothelial-cadherin
FITC	fluorescein isothiocyanate

## References

[1] Yamagata K., Matsumura K., Inoue W., Shiraki T., Suzuki K., Yasuda S., Sugiura H., Cao C., Watanabe Y., Kobayashi S. (2001). Coexpression of microsomal-type prostaglandin E synthase with cyclooxygenase-2 in brain endothelial cells of rats during endotoxin-induced fever. J. Neurosci..

[2] Takemiya T., Matsumura K., Sugiura H., Maehara M., Yasuda S., Uematsu S., Akira S., Yamagata K. (2010). Endothelial microsomal prostaglandin E synthase-1 exacerbates neuronal loss induced by kainate. J. Neurosci. Res..

[3] Rose J.W., Hill K.E., Watt H.E., Carlson N.G. (2004). Inflammatory cell expression of cyclooxygenase-2 in the multiple sclerosis lesion. J. Neuroimmunol..

[4] Deininger M.H., Schluesener H.J. (1999). Cyclooxygenases-1 and -2 are differentially localized to microglia and endothelium in rat EAE and glioma. J. Neuroimmunol..

[5] Mendel I., Kerlero de Rosbo N., Ben-Nun A. (1995). A myelin oligodendrocyte glycoprotein peptide induces typical chronic experimental autoimmune encephalomyelitis in H-2^b^ mice: Fine specificity and T cell receptor Vβ expression of encephalitogenic T cells. Eur. J. Immunol..

[6] Kuerten S., Kostova-Bales D.A., Frenzel L.P., Tigno J.T., Tary-Lehmann M., Angelov D.N., Lehmann P.V. (2007). MP4- and MOG:35-55-induced EAE in C57BL/6 mice differentially targets brain, spinal cord and cerebellum. J. Neuroimmunol..

[7] Bolton C., Gordon D., Turk J.L. (1984). Prostaglandin and thromboxane levels in central nervous system tissues from rats during the induction and development of experimental allergic encephalomyelitis (EAE). Immunopharmacology.

[8] Marusic S., Leach M.W., Pelker J.W., Azoitei M.L., Uozumi N., Cui J., Shen M.W., DeClercq C.M., Miyashiro J.S., Carito B.A. (2005). Cytosolic phospholipase A2 alpha-deficient mice are resistant to experimental autoimmune encephalomyelitis. J. Exp. Med..

[9] Kihara Y., Matsushita T., Kita Y., Uematsu S., Akira S., Kira J., Ishii S., Shimizu T. (2009). Targeted lipidomics reveals mPGES-1-PGE2 as a therapeutic target for multiple sclerosis. Proc. Natl. Acad. Sci. USA.

[10] Takeuchi C., Matsumoto Y., Kohyama K., Uematsu S., Akira S., Yamagata K., Takemiya T. (2013). Microsomal prostaglandin E synthase-1 aggravates inflammation and demyelination in a mouse model of multiple sclerosis. Neurochem. Int..

[11] Miyamoto K., Miyake S., Mizuno M., Oka N., Kusunoki S., Yamamura T. (2006). Selective COX-2 inhibitor celecoxib prevents experimental autoimmune encephalomyelitis through COX-2-independent pathway. Brain.

[12] Muthian G., Raikwar H.P., Johnson C., Rajasingh J., Kalgutkar A., Marnett L.J., Bright J.J. (2006). COX-2 inhibitors modulate IL-12 signaling through JAK-STAT pathway leading to Th1 response in experimental allergic encephalomyelitis. J. Clin. Immunol..

[13] Ni J., Shu Y.Y., Zhu Y.N., Fu Y.F., Tang W., Zhong X.G., Wang H., Yang Y.F., Ren J., Wang M.W. (2007). COX-2 inhibitors ameliorate experimental autoimmune encephalomyelitis through modulating IFN-gamma and IL-10 production by inhibiting T-bet expression. J. Neuroimmunol..

[14] Martin D., Near S.L. (1995). Protective effect of the interleukin-1 receptor antagonist (IL-1RA) on experimental allergic encephalomyelitis in rats. J. Neuroimmunol..

[15] Schiffenbauer J., Streit W.J., Butfiloski E., LaBow M., Edwards C., Moldawer L.L. (2000). The induction of EAE is only partially dependent on TNF receptor signaling but requires the IL-1 type I receptor. Clin. Immunol..

[16] Takemiya T., Takeuchi C., Kawakami M. (2017). Microsomal prostaglandin E synthase-1 facilitates an intercellular interaction between CD4(+) T cells through IL-1beta autocrine function in experimental autoimmune encephalomyelitis. Int. J. Mol. Sci..

[17] Rosenberg G.A. (2002). Matrix Metalloproteinases in Neuroinflammation. Glia.

[18] Mun-Bryce S., Rosenberg G.A. (1998). Gelatinase B modulates selective opening of the blood-brain barrier during inflammation. Am. J. Physiol..

[19] Madri J.A., Graesser D., Haas T. (1996). The roles of adhesion molecules and proteinases in lymphocyte transendothelial migration. Biochem. Cell Biol..

[20] Seabrook T.J., Littlewood-Evans A., Brinkmann V., Pollinger B., Schnell C., Hiestand P.C. (2010). Angiogenesis is present in experimental autoimmune encephalomyelitis and pro-angiogenic factors are increased in multiple sclerosis lesions. J. Neuroinflamm..

[21] Rigau V., Morin M., Rousset M.C., de Bock F., Lebrun A., Coubes P., Picot M.C., Baldy-Moulinier M., Bockaert J., Crespel A. (2007). Angiogenesis is associated with blood-brain barrier permeability in temporal lobe epilepsy. Brain.

[22] Ortiz G.G., Pacheco-Moises F.P., Macias-Islas M.A., Flores-Alvarado L.J., Mireles-Ramirez M.A., Gonzalez-Renovato E.D., Hernandez-Navarro V.E., Sanchez-Lopez A.L., Alatorre-Jimenez M.A. (2014). Role of the blood-brain barrier in multiple sclerosis. Arch. Med. Res..

[23] Roscoe W.A., Welsh M.E., Carter D.E., Karlik S.J. (2009). VEGF and angiogenesis in acute and chronic MOG(35-55) peptide induced EAE. J. Neuroimmunol..

[24] Muramatsu R., Takahashi C., Miyake S., Fujimura H., Mochizuki H., Yamashita T. (2012). Angiogenesis induced by CNS inflammation promotes neuronal remodeling through vessel-derived prostacyclin. Nat. Med..

[25] Brumm A.J., Carmichael S.T. (2012). Not just a rush of blood to the head. Nat. Med..

[26] Dore-Duffy P., Donaldson J.O., Koff T., Longo M., Perry W. (1986). Prostaglandin release in multiple sclerosis: Correlation with disease activity. Neurology.

[27] Aberg J.A., Demers L.M., Romano P.J., Tenser R.B. (1990). Prostaglandin production in chronic progressive multiple sclerosis. J. Clin. Lab. Anal..

[28] Jakobsson P.J., Thoren S., Morgenstern R., Samuelsson B. (1999). Identification of human prostaglandin E synthase: A microsomal, glutathione-dependent, inducible enzyme, constituting a potential novel drug target. Proc. Natl. Acad. Sci. USA.

[29] Tanikawa N., Ohmiya Y., Ohkubo H., Hashimoto K., Kangawa K., Kojima M., Ito S., Watanabe K. (2002). Identification and characterization of a novel type of membrane-associated prostaglandin E synthase. Biochem. Biophys. Res. Commun..

[30] Tanioka T., Nakatani Y., Semmyo N., Murakami M., Kudo I. (2000). Molecular identification of cytosolic prostaglandin E2 synthase that is functionally coupled with cyclooxygenase-1 in immediate prostaglandin E2 biosynthesis. J. Biol. Chem..

[31] Murakami M., Naraba H., Tanioka T., Semmyo N., Nakatani Y., Kojima F., Ikeda T., Fueki M., Ueno A., Oh S. (2000). Regulation of prostaglandin E2 biosynthesis by inducible membrane-associated prostaglandin E2 synthase that acts in concert with cyclooxygenase-2. J. Biol. Chem..

[32] Taniguchi H., Anacker C., Suarez-Mier G.B., Wang Q., Andreasson K. (2011). Function of prostaglandin E2 EP receptors in the acute outcome of rodent hypoxic ischemic encephalopathy. Neurosci. Lett..

[33] Frankowski J.C., DeMars K.M., Ahmad A.S., Hawkins K.E., Yang C., Leclerc J.L., Dore S., Candelario-Jalil E. (2015). Detrimental role of the EP1 prostanoid receptor in blood-brain barrier damage following experimental ischemic stroke. Sci. Rep..

[34] Omori K., Kida T., Hori M., Ozaki H., Murata T. (2014). Multiple roles of the PGE2 -EP receptor signal in vascular permeability. Br. J. Pharmacol..

[35] Dalvi S., Nguyen H.H., On N., Mitchell R.W., Aukema H.M., Miller D.W., Hatch G.M. (2015). Exogenous arachidonic acid mediates permeability of human brain microvessel endothelial cells through prostaglandin E2 activation of EP3 and EP4 receptors. J. Neurochem..

[36] Liang X., Lin L., Woodling N.S., Wang Q., Anacker C., Pan T., Merchant M., Andreasson K. (2011). Signaling via the prostaglandin E(2) receptor EP4 exerts neuronal and vascular protection in a mouse model of cerebral ischemia. J. Clin. Investig..

[37] Levesque S.A., Pare A., Mailhot B., Bellver-Landete V., Kebir H., Lecuyer M.A., Alvarez J.I., Prat A., de Rivero Vaccari J.P., Keane R.W. (2016). Myeloid cell transmigration across the CNS vasculature triggers IL-1beta-driven neuroinflammation during autoimmune encephalomyelitis in mice. J. Exp. Med..

[38] Blamire A.M., Anthony D.C., Rajagopalan B., Sibson N.R., Perry V.H., Styles P. (2000). Interleukin-1beta-induced changes in blood-brain barrier permeability, apparent diffusion coefficient, and cerebral blood volume in the rat brain: A magnetic resonance study. J. Neurosci..

[39] Argaw A.T., Zhang Y., Snyder B.J., Zhao M.L., Kopp N., Lee S.C., Raine C.S., Brosnan C.F., John G.R. (2006). IL-1β regulates blood-brain barrier permeability via reactivation of the hypoxia-angiogenesis program. J. Immunol..

[40] Correale J., Farez M.F. (2015). The role of astrocytes in multiple sclerosis progression. Front. Neurol..

[41] Wang Y., Jin S., Sonobe Y., Cheng Y., Horiuchi H., Parajuli B., Kawanokuchi J., Mizuno T., Takeuchi H., Suzumura A. (2014). Interleukin-1beta induces blood-brain barrier disruption by downregulating sonic hedgehog in astrocytes. PLoS ONE.

[42] Murta V., Farias M.I., Pitossi F.J., Ferrari C.C. (2015). Chronic systemic IL-1beta exacerbates central neuroinflammation independently of the blood-brain barrier integrity. J. Neuroimmunol..

[43] Vainchtein I.D., Vinet J., Brouwer N., Brendecke S., Biagini G., Biber K., Boddeke H.W., Eggen B.J. (2014). In acute experimental autoimmune encephalomyelitis, infiltrating macrophages are immune activated, whereas microglia remain immune suppressed. Glia.

[44] Dumas A., Amiable N., de Rivero Vaccari J.P., Chae J.J., Keane R.W., Lacroix S., Vallieres L. (2014). The inflammasome pyrin contributes to pertussis toxin-induced IL-1beta synthesis, neutrophil intravascular crawling and autoimmune encephalomyelitis. PLoS Pathog..

[45] McFarland H.F., Martin R. (2007). Multiple sclerosis: A complicated picture of autoimmunity. Nat. Immunol..

[46] Martin B.N., Wang C., Zhang C.J., Kang Z., Gulen M.F., Zepp J.A., Zhao J., Bian G., Do J.S., Min B. (2016). T cell-intrinsic ASC critically promotes T(h)17-mediated experimental autoimmune encephalomyelitis. Nat. Immunol..

[47] Okano M., Sugata Y., Fujiwara T., Matsumoto R., Nishibori M., Shimizu K., Maeda M., Kimura Y., Kariya S., Hattori H. (2006). E prostanoid 2 (EP2)/EP4-mediated suppression of antigen-specific human T-cell responses by prostaglandin E2. Immunology.

[48] Maslanka T., Spodniewska A., Barski D., Jasiecka A., Zuska-Prot M., Ziolkowski H., Markiewicz W., Jaroszewski J.J. (2014). Prostaglandin E(2) down-regulates the expression of CD25 on bovine T cells, and this effect is mediated through the EP4 receptor. Vet. Immunol. Immunopathol..

[49] Sreeramkumar V., Hons M., Punzon C., Stein J.V., Sancho D., Fresno M., Cuesta N. (2016). Efficient T-cell priming and activation requires signaling through prostaglandin E2 (EP) receptors. Immunol. Cell Biol..

[50] Zhu P., Genc A., Zhang X., Zhang J., Bazan N.G., Chen C. (2005). Heterogeneous expression and regulation of hippocampal prostaglandin E2 receptors. J. Neurosci. Res..

[51] Cao C., Matsumura K., Yamagata K., Watanabe Y. (1996). Endothelial cells of the rat brain vasculature express cyclooxygenase-2 mRNA in response to systemic interleukin-1 beta: A possible site of prostaglandin synthesis responsible for fever. Brain Res..

[52] Serou M.J., DeCoster M.A., Bazan N.G. (1999). Interleukin-1β activates expression of cyclooxygenase-2 and inducible nitric oxide synthase in primary hippocampal neuronal culture: Platelet-activating factor as a preferential mediator of cyclooxygenase-2 expression. J. Neurosci. Res..

[53] Samad T.A., Moore K.A., Sapirstein A., Billet S., Allchorne A., Poole S., Bonventre J.V., Woolf C.J. (2001). Interleukin-1β-mediated induction of COX-2 in the CNS contributes to inflammatory pain hypersensitivity. Nature.

[54] Laflamme N., Lacroix S., Rivest S. (1999). An essential role of interleukin-1beta in mediating NF-kappaB activity and COX-2 transcription in cells of the blood-brain barrier in response to a systemic and localized inflammation but not during endotoxemia. J. Neurosci..

[55] Favrais G., Schwendimann L., Gressens P., Lelievre V. (2007). Cyclooxygenase-2 mediates the sensitizing effects of systemic IL-1-beta on excitotoxic brain lesions in newborn mice. Neurobiol. Dis..

[56] Casos K., Siguero L., Fernandez-Figueras M.T., Leon X., Sarda M.P., Vila L., Camacho M. (2011). Tumor cells induce COX-2 and mPGES-1 expression in microvascular endothelial cells mainly by means of IL-1 receptor activation. Microvasc. Res..

[57] Van Dam A.M., De Vries H.E., Kuiper J., Zijlstra F.J., De Boer A.G., Tilders F.J., Berkenbosch F. (1996). Interleukin-1 receptors on rat brain endothelial cells: A role in neuroimmune interaction?. FASEB J..

[58] Wohleb E.S., Patterson J.M., Sharma V., Quan N., Godbout J.P., Sheridan J.F. (2014). Knockdown of interleukin-1 receptor type-1 on endothelial cells attenuated stress-induced neuroinflammation and prevented anxiety-like behavior. J. Neurosci..

[59] Li Q., Powell N., Zhang H., Belevych N., Ching S., Chen Q., Sheridan J., Whitacre C., Quan N. (2011). Endothelial IL-1R1 is a critical mediator of EAE pathogenesis. Brain. Behav. Immun..

[60] McCandless E.E., Budde M., Lees J.R., Dorsey D., Lyng E., Klein R.S. (2009). Il-1r signaling within the central nervous system regulates CXCL12 expression at the blood-brain barrier and disease severity during experimental autoimmune encephalomyelitis. J. Immunol..

[61] Uematsu S., Matsumoto M., Takeda K., Akira S. (2002). Lipopolysaccharide-dependent prostaglandin E(2) production is regulated by the glutathione-dependent prostaglandin E(2) synthase gene induced by the Toll-like receptor 4/MYD88/NF-IL6 pathway. J. Immunol..

[62] Sakuma H., Kohyama K., Park I.K., Miyakoshi A., Tanuma N., Matsumoto Y. (2004). Clinicopathological study of a myelin oligodendrocyte glycoprotein-induced demyelinating disease in LEW.1AV1 rats. Brain.

[63] Storch M.K., Stefferl A., Brehm U., Weissert R., Wallstrom E., Kerschensteiner M., Olsson T., Linington C., Lassmann H. (1998). Autoimmunity to myelin oligodendrocyte glycoprotein in rats mimics the spectrum of multiple sclerosis pathology. Brain Pathol..

