# Supplementary material for: Endothelial Microsomal Prostaglandin E Synthetase-1 Upregulates Vascularity and Endothelial Interleukin-1β in Deteriorative Progression of Experimental Autoimmune Encephalomyelitis
Source: Int J Mol Sci. 2018 Nov 19;19(11):3647. doi: 10.3390/ijms19113647 (PMC6274996; doi:10.3390/ijms19113647)
Supplement: Supplementary file 1 [file ijms-19-03647-s001.zip › Supplementary Figure 1.docx]

**
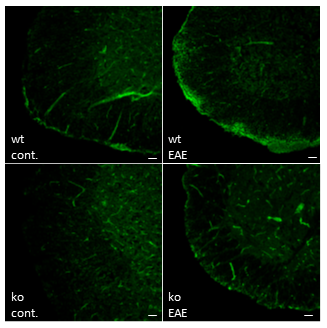
Supplementary Figure 1.** Distribution of tomato lectin staining in spinal cords. Immunohistochemistry image showing tomato lectin in the spinal cord in wild-type (wt) control, wt experimental autoimmune encephalomyelitis (EAE), microsomal PGE synthase-1-deficient (*mPGES-1^-/-^*) control, and *mPGES-1^-/-^* EAE mice. Scale bar (50 μm). wt, wild-type; ko, *mPGES-1^-/-^*; cont., naïve mice; EAE, EAE mice.
